# Review on Recent Advance of 3DP-Based Pediatric Drug Formulations

**DOI:** 10.1155/2024/4875984

**Published:** 2024-09-26

**Authors:** Aychew Mekuriaw Tegegne, Kassahun Dires Ayenew, Muluken Nigatu Selam

**Affiliations:** ^1^ Department of Pharmacy Medicine and Health Science College Debre Berhan University, Debre Berhan, Ethiopia; ^2^ Department of Pharmaceutics and Social Pharmacy School of Pharmacy College of Health Sciences Addis Ababa University, Addis Ababa, Ethiopia

**Keywords:** 3DP technology, dosage form, individualized therapy, pediatric formulation

## Abstract

Three-dimensional printing (3DP) has emerged as a game-changing technology in the pharmaceutical industry, providing novel formulation development in the pharmaceutical sector as a whole, which improved patients' individualized therapy. The pediatric population is among the key targets for individualized therapy. Children are a diverse group that includes neonates, infants, and toddlers, each with unique physiological characteristics. Treatment adherence has a significant impact on safe and effective pharmacotherapy in the pediatric population. Improvement of therapeutic dosage forms that provide for the special demands of the pediatric population is a significant challenge for the pharmaceutical industry. Scientists have actively explored 3DP, a quick prototype manufacturing method that has emerged in recent years from many occupations due to its benefits of modest operation, excellent reproducibility, and vast adaptability. This review illuminates the most widely used 3DP technology and its application in the development of pediatric-friendly drug formulations. This 3DP technology allows optimization of pediatric dosage regimens and cases that require individualized treatment, such as geriatrics, renal impairment, liver impairment, critically ill, pregnancy populations, and drugs with nonlinear pharmacokinetics. The fast evolution of 3DP expertise, in addition to the introduction of pharmaceutical inks, has enormous promise for patient dosage form customization.

## 1. Introduction

Over the last 10 years, there has been a movement in attitudes toward pharmacological therapy approaches. Efforts are being made by researchers to place a greater emphasis on patients' genetic and physiological characteristics in order to tailor their pharmacological therapy. By adopting the notion of personalized medicine, the achievement of medical treatment may be improved while substantially lowering the threat of bad effects, which can occasionally lead to life-threatening situations [1].

The pediatric population is among the key targets of individualized therapy [2]. Children are a diverse group that includes neonates, babies, and toddlers, each with unique physiological characteristics. A child's physical growth happens swiftly throughout the first few months of life. Drug medication must be regularly adjusted due to the fast development and maturation [3]. Treatment adherence has a significant impact on safe and effective pharmacotherapy in the pediatric population, in which formulation palatability and caregiver constancy who administer the formulation are the key contributing elements [4]. Organoleptic qualities such as smell, taste, texture, vision, and perhaps sound are important factors for the creation of pediatric dosage forms, and their significance has been acknowledged by both the pharmaceutical industry and regulatory organizations [5]. In recent research with 153 pediatric participants, the top cause for a patient's unwillingness to take the provided medication was the taste of the formulations [6]. It should be noted that pediatric patients are still utilizing medications that were primarily developed for adults. These drugs are utilized secondhand to treat children because personalized treatments are not available in most instances [7].

The improvement of therapeutic dosage forms to accommodate the special demands of the pediatric population is a significant challenge for the pharmaceutical industry. Often, youngsters are unable to swallow regular tablets or capsules. Healthcare experts often ban manipulation techniques such as splitting tablets or opening capsules, as they may result in the administration of incorrect amounts. Furthermore, the classic approaches described above have certain limitations in terms of dosage modification. These restrictions are mostly demonstrated in three ways, as follows: First, there are just a few types of formulations suitable for therapeutically split dosages. Most available dosage forms within the pharmaceutical sector, such as coated formulations, sustained and controlled release formulations, and osmotic pump formulations, are not suitable for use in divided doses; otherwise, the drug release characteristics will be affected, and the tablet's stability will be compromised. Opening the original packaging of products and affecting the integrity of the tablets through the cutting process may lead to some tablets being exposed to moisture and oxidation, which may result in unwanted consequences on the tablets' quality and the operator's health [8]. Some very hazardous medications, such as anticancer treatments, produce powder when split or crushed, which might be harmful to the operator's health [9].

As a result, scientific dosage division or the development of exact alternative dose division procedures can significantly improve children's practical medication levels. Three-dimensional printing (3DP), often referred to as additive manufacturing, is a method in which a solid item is produced by stacking deposits of material on top of each other layer by layer [10, 11]. The term 3DP refers to a variety of 3DP technologies that differ in their beginning materials and printers. Although 3DP is still a relatively new production technology, it has already been adopted by the pharmaceutical sector. The development process begins with the compilation of a digital dossier that contains the particular geometry of the object to be printed. The digital file can be modified to change the shape or size of the final product [12]. It is a quick prototype manufacturing method that emerged in recent years and has been actively explored by scientists from many walks of life due to its benefits of modest operation, excellent reproducibility, and vast adaptability [13, 14]. Several publications have demonstrated the usefulness of 3DP in dose management [15], medicine combination, and recommended modifications to pediatric-friendly forms [8, 16, 17]. Fused deposition modeling (FDM) 3DP has been utilized to build child-friendly forms and to provide better flavor masking [16].

The objective of this review is to illuminate the most widely used 3DP technology and its recent applications in the development of pediatric-friendly drug formulations.

## 2. 3DP and Pharmaceuticals

A 3DP has emerged as a game-changing knowledge in the pharmaceutical business, providing novel solutions for patients and the sector as a whole. This technology makes it possible for the on-demand design and production of individualized medicinal products, resulting in customized doses, medication combinations, and release patterns. It also enables the development of pharmaceuticals with unique forms, sizes, and textures, which might benefit patients, pharmacists, and the pharmaceutical business. Several firms and academic organizations are investigating the applications of 3DP in pharmaceuticals, which has the possibility of indicating how medications are created and delivered [18].

The pharmaceutical sector is becoming increasingly interested in 3DP's ability to generate personalized medications customized to a patient's individual needs. This technique has benefits such as environmentally friendly production, reduced medication and excipient waste, and the opportunity to produce formulations with tailored drug release patterns and multidrug combinations. Furthermore, 3DP can simplify the small-scale manufacture of pharmaceuticals, which is very useful for clinical trials and customized medicine [18].

Several firms are pioneering the utilization of 3DP to create customized medications. Triastek Inc., for example, is the world's pioneer in pharmaceutical 3DP, employing its unique MED 3DP technology to create customizable tablets in various forms and geometries that manage medication onset time, duration, and interactions with the body. Aprecia Pharmaceuticals, GlaxoSmithKline (GSK), and Fabrication Pharmacy (FabRx) are actively working on the research and marketing of 3D-printed medications for various medical diseases [19].

Despite the potential benefits of 3DP in medicines, there are several hurdles to overcome, such as regulatory restrictions, evidence-based safety and efficacy, and the development of pharmaceutically relevant 3DP technologies. However, great progress has been achieved in overcoming these problems, and the integration of 3DP into the pharmaceutical industry is an area of active development [20].

A 3DP technology is transforming the pharmaceutical sector by allowing for on-demand manufacture of tailored medicinal items. This technique has the possibility of enhancing patient outcomes, decreasing medication waste, and allowing the creation of individualized medications for specific medical requirements. While there are obstacles to overcome, current research and development in this discipline show much promise for the future of pharmaceutical manufacture and customized treatment [21].

### 2.1. Pharmaceutical Application of 3DP

#### 2.1.1. Drug Formulation and Development

Thanks to the ability to create individualized formulations based on the needs of each patient, 3DP allows for exact control over medication dose and release patterns. Complex drug designs, such as multilayered tablets with variable release rates, may be produced because of this technique [19, 22]. Drug development frequently faces difficulties due to poor water solubility, which can restrict the bioavailability and effectiveness of some drugs. By creating medications in innovative dosage forms that improve solubility and dissolving rates, 3DP presents the potential to get around this restriction [23]. The production of amorphous solid dispersions or solid solutions using methods like hot melt extrusion (HME) in conjunction with 3DP might increase the bioavailability of poorly soluble medications [24]. Combination medicines are frequently used to treat several disease pathways at once or complicated medical disorders. The creation of dosage forms with several pharmaceuticals in precise ratios is made possible by 3DP, which makes it easier to create personalized combination goods. This strategy can lessen the number of tablets needed to manage several drugs, increase patient adherence, and improve treatment results [24].

#### 2.1.2. Personalized Medicine

3DP makes it easier to create customized medicine doses and delivery systems by using patient-specific data, such as genetic and medical imaging. This personalization reduces side effects and increases therapeutic efficacy. 3DP enables the creation of complex dosage forms, such as tablets, capsules, and implants. Because of its adaptability, innovative drug delivery systems that are tailored for certain therapeutic purposes can be developed [22, 25]. Patients' reactions to drugs might differ greatly because of things like age, weight, metabolism, and genetic composition. Customized medicine dose forms that precisely fit each patient's dosage requirements may be made thanks to 3DP [26].

Beyond customizable dose forms, 3DP enables the production of unique medication delivery systems that are matched to each patient's particular anatomical and physiological traits. To ensure the best possible medication deposition in the lungs, inhalers, for instance, can be made to fit each patient's unique airflow patterns and inhaling capacity. In a similar vein, transdermal patches may be tailored to conform well to certain body parts, improving medication absorption and patient compliance [27]. The discovery of genetic differences that impact an individual's reaction to particular drugs has been made possible by advancements in the fields of genomics and pharmacogenomics. Healthcare practitioners are able to create individualized treatment plans based on a patient's genetic profile by combining 3DP technology with data from genetic testing. For instance, medication formulations can be customized to each patient's specific metabolic pathways and levels of enzyme activity, maximizing therapeutic efficacy while lowering the possibility of unfavorable drug responses [27].

#### 2.1.3. Drug Delivery Devices

Complex drug delivery devices with specialized forms, sizes, and release mechanisms, such as transdermal patches, implants, and inhalers, may be made thanks to 3DP. These gadgets improve therapeutic results, patient compliance, and medication efficacy.

##### 2.1.3.1. Biofabrication of Tissues and Organs

Using bioink made of cells, growth factors, and biomaterials, 3D bioprinting in regenerative medicine allows the creation of live tissues and organs. Applications such as tissue engineering, organ transplantation, and drug testing show promise for this technique [28]. When it comes to creating drug delivery devices with intricate geometries, conventional production methods may have limits. Using traditional methods, it is difficult or impossible to produce elaborate structures and features. However, 3DP makes this feasible. Devices with detailed drug reservoirs, exact microfluidic channels, or customized geometries based on the anatomy of each patient fall under this category [29].

Customizing devices to meet each patient's unique demands is one of the main benefits of 3DP in medication delivery systems. The creation of combination devices that administer numerous medications or therapies concurrently is made possible by the ability to integrate many components into a single drug delivery device through the use of 3DP. Inhalers, for instance, can be made to provide a mix of corticosteroids and bronchodilators to treat asthma or chronic obstructive pulmonary disease (COPD) [30]. Likewise, it is possible to create implanted devices that deliver many medications at different times in precisely calibrated dosages. The release properties of drug-loaded implants, microparticles, or other dosage forms may be precisely tuned by researchers by varying parameters, including material composition, porosity, and shape. Drug delivery device prototypes and iterative designs are sped up by 3DP, enabling researchers to test various configurations, analyze performance metrics, and iterate designs more quickly. Faster development schedules and more effective optimization and implementation of functionality and performance are made possible by this quick iteration [31].

#### 2.1.4. Formulation Optimization

Because 3DP makes it possible to fabricate small-scale dose forms for preclinical testing, it speeds up the process of fast prototyping and iterative optimization of medication formulations. This shortens the time it takes for new pharmaceutical products to reach the market and speeds up the medication development process. The capacity to quickly model and test novel formulations may be hampered by the time-consuming procedures associated with traditional formulation development, such as tableting or encapsulation. Dosage forms may be quickly created with exact control over shape, composition, and drug release kinetics. This speeds up the entire development process by allowing researchers to test several formulas and iterate on ideas quickly [32, 33].

Researchers can find the best formulations with enhanced bioavailability, solubility, or dissolving properties by methodically adjusting these parameters and assessing their impact on drug release, stability, and pharmacokinetics. The use of 3DP makes it possible to create complicated medication compositions that would be difficult to make using conventional techniques [33]. Using 3DP technology, for instance, it is simple to make combination products with several active pharmacological ingredients (APIs) or multilayered tablets with distinct drug release characteristics. This makes it possible for scientists to investigate new formulation techniques and enhance medication delivery methods for certain therapeutic uses. Small-scale manufacturing of dosage forms for preclinical testing and assessment is made easier by 3DP technology [34].

Without the need for large-scale production facilities, researchers may create specially formulated dosage forms suited to the requirements of preclinical investigations, such as animal models or in vitro experiments [34]. This adaptability lowers the resources needed for early-stage research and enables more effective screening of formulation candidates. Formulations that are patient-centric and customized to meet each patient's requirements and preferences can be created thanks to 3DP. Through the integration of patient-specific data, such as pharmacogenomics or medical imaging, scientists may develop customized dosage forms that are optimized for many aspects, such as drug release kinetics, administration route, and dosing schedule [32]. Treatment adherence, patient happiness, and therapeutic results are all enhanced by this individualized approach. Formulation optimization can benefit from a variety of additive manufacturing processes, including selective laser sintering (SLS), stereolithography (SLA), and FDM. For instance, SLA provides the high resolution and precision production of complex structures, whereas FDM permits the exact deposition of drug-loaded filaments to construct dosage forms with controlled drug release patterns. These methods can be used by researchers to investigate novel formulation strategies and create cutting-edge medication delivery systems [18].

#### 2.1.5. Dose Combination Products

Combination therapy for difficult medical diseases is made possible by the simultaneous manufacture of many medications within a single dosage form made possible by 3DP. This method increases patient adherence, lowers the number of pills taken, and improves therapy efficacy. With the use of 3DP technology, many medications may be combined into a single dosage form to create fixed-dose combination medicines that enhance treatment adherence and streamline medication administration. Fixed-dose combination solutions can decrease the risk of medication mistakes and adverse drug interactions while increasing treatment efficacy by combining complementary treatments or addressing numerous disease pathways at once [35].

Complicated dosage forms with several layers or compartments, each holding a distinct medication or release profile, may be created with 3DP. To maximize absorption and reduce gastrointestinal side effects, stacked tablets, for instance, might be made to release medications in a sequential manner at various points throughout the gastrointestinal system. Comparably, medications with various pharmacokinetic properties can be delivered using multicompartment dosage forms, enabling customized dosing schedules and better therapeutic results. The pharmaceutical business might undergo significant transformations with the help of 3DP, ranging from personalized medicine and regenerative treatments to drug research and production. Technology is predicted to become more and more important in determining the direction of healthcare as it develops [20].

## 3. 3DP Technology

**Figure 1:**
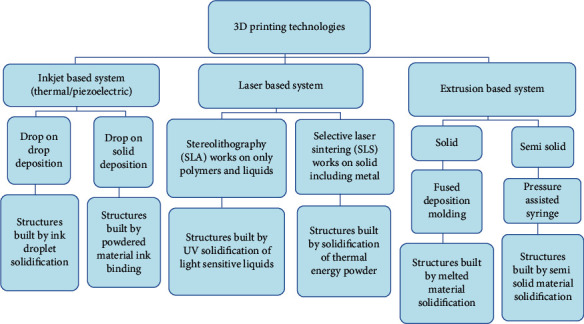
Classification of the main 3DP technologies.

A 3DP, often referred to as additive manufacturing, is a highly intelligent technique that produces 3-dimensional items by depositing successive layers of the employed material under the supervision of computer software. Its capacity to manufacture multiple forms and geometries (Table 1) remains one of its most significant production benefits. It has its roots in engineering and several nonmedical activities, particularly in the car industry. Recently used in medical devices [42], implants [43], and pharmaceutical dosage forms [44] have been proven with a sense of enthusiasm, particularly in expressions of their potential in customized treatment. Several printing methods have been tried for the manufacture of medicinal items throughout the last decade [45]. The introduction and evolution of 3DP technology began with the widely used inkjet printing (IJP) method, which was then expanded with additional printing technologies. Recently, two innovative 3DP methods such as stencil printing [46] and embedded 3DP [47] have been evaluated for the viability of developing novel medications that are appropriate for tailored therapy and will be more useful to pediatric populations.  Figure 1 highlights the categorization and the main mechanisms used by the most prevalent 3DP methods [48].

### 3.1. IJP

**Figure 2:**
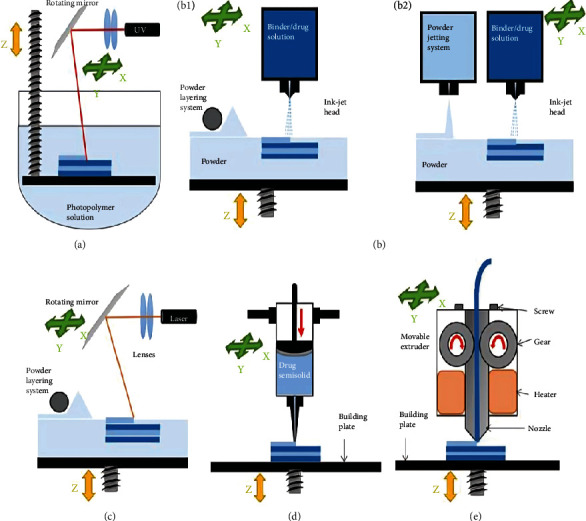
Mechanisms of different 3DP technologies. (a) Stereolithographic (SLA), (b) (b1, b2) powder bed and powder jetting, (c) selective laser sintering (SLS), (d) semisolid extrusion (SSE), and (e) fused deposition modeling (FDM) [49].

The powder bed (PB) inkjet 3DP technique has been extensively employed to create many sorts of very porous 3D items. A schematic representation of the inkjet 3DP process is presented in Figures 2(b1) and 2(b2). The binder used in this approach is dictated by the material's composition. The 3D additive technique machinery may be applied to powder-based materials with the appropriate particle size/shape and followability. To ensure efficient printing, starting materials should have particle size <1 *μ*m, viscosity <20 cP, and surface tension ~50 mN/m. Bonding mechanism, conversion from amorphous to crystallization, anhydrous to hydrous, and polymorphism modifications are all expected to occur during the wet granulation process, depending on the kind of solvent employed and the physicochemical qualities of the powders. In industrial IJP, print heads are classified into two types: continuous inkjet and drop-on-demand. The inkjet material deposition technology commonly employs piezoelectric print heads to create several layers on substrates' surfaces. Compared to thermal print heads, piezoelectric printing heads exhibit superior control over droplet generation and are hence preferable for use in product development. Piezoelectric print heads may employ a variety of inks, whereas thermal heads are confined to volatile solvents. DoD IJP is said to be more precise, but it also uses less ink to save waste. With the development of fast-curing ultraviolet light-emitting diode (UV-LED)–based inks, affordable DoD technology offers numerous designs that will most likely fulfill the rising demands of product printing, which is kid-friendly and suited for customized choice [50].

### 3.2. FDM

FDM, a sort of 3D extrusion technique, has been intensively researched for pharmaceutical applications.  Figure 2(e) illustrates a schematic illustration of the FDM technique. In fused filament fabrication (FFF) modeling, the thermoplastic polymeric filament is fed continuously through a heated nozzle head before melting just over the glass transition temperature. To construct a desirable 3D item, extruded fused material is put successively in three dimensions onto a substrate and cooled. Extrusion printing uses a variety of materials including pastes, polymers, silicones, suspensions, and other semisolids. Solid polymeric filaments, on the other hand, are used in FFF to print scaffolds, drug-eluting devices, and geometrically structured drug delivery systems with altered or customized release profiles [41].

HME may be used to continuously produce filament from coiled thermoplastic polymers for use in FDM printing. Polylactic acid (PLA), polyvinyl acetate (PVA), acrylonitrile butadiene styrene (ABS), and ethylene vinyl acetate (EVA) are all thermoplastic polymers that are generally considered safe [41]. The feedstock filaments in combination with active pharmaceutical ingredients are based on the unique characteristics of the polymers, such as insolubility (PVA; Eudragit RL), fast dissolution (polyvinylpyrrolidone), enteric (Eudragit L30D55), and swellable/erodible (hydroxypropyl methylcellulose [HPMC]), and were appropriately utilized for printing capsules or any other dosage forms for instantaneous release or modified release dosage forms. Another advantage of utilizing HME is that it allows the inclusion of a homogeneous solid dispersion of thermostable drug(s) and/or excipients in the filament components as printable material, which is not possible using other technologies. The integrated method provides an automated production process with great efficiency and little material loss [51]. The versatility of FDM in altering medication loading and release patterns by shifting the percentage content of feedstock or modifying the geometric shape makes it an appealing solution for formulation scientists to create pediatric-friendly products depending on patient preferences. A recent attempt was made to build a unified equipment certification framework for FDM printers, which is vital to speed and streamline the approval process for developing 3D-printed items [52].  Figure 2 shows the mechanisms of different 3DP technologies [49].

### 3.3. Semisolid Extrusion System

The pressure-assisted microsyringe (PAM) printing technique involves the semisolid material extrusion from syringes at pressures ranging from three to five bars to build the required 3D structure.  Figure 2(d) displays a schematic illustration of the semisolid extrusion system 3DP process. This approach is commonly used to create scaffolds in tissue engineering and the field of regenerative medicine applications. The building of the 3D heterogeneous scaffolds' supporting framework for the transportation of proteins and growth factors has been proven using multisyringe PAMs [40].

Extrusion 3DP is useful since it eliminates the need for high temperatures during fabrication. As a result, product distortion after drying may result in porous structures that disintegrate quickly. A compressed air pressure, piston, or extruder is used to expel a semisolid or semimolten substance via a micrometer hole. Gel and paste with the proper viscosity can be applied as a starting material to construct drug delivery systems with distinct release profiles. The semisolid extrusion process is a low-resolution method where the 3DP orifice diameter is rather large (0.4–0.8 mm), and the final product may not be able to support the weight of the layers that came before it [53].

The dual-head extrusion technique can handle two substances with disparate melting points. This approach produced delayed-release theophylline tablets with an inner layer of polyvinylpyrrolidone and an enteric layer of Eudragit L with a thickness of more than 0.52 mm to provide appropriate acid protection [54]. The dual-head printer was also employed to manufacture two separate compartment tubes made of insoluble PLA polymer and then cap-sealed with PVA. The authors described 3D-printed capsular devices with several compartments for pulsed drug administration. The development of such adaptable drug delivery systems is interesting because it might customize customized therapy with numerous combinations of drugs with diverse release rates and exact dosage modification based on their fill quantity [37].

### 3.4. SLS 3DP

An SLS 3DP is comparable to PB 3DP; however, in this case, laser radiations are utilized to liquefy (totally or partly) and fuse the stacked powders. This is a typical approach for 3D metal printing [55]. Powders or beginning materials that might be utilized include polyamides, polystyrenes, and polycarbonates. The sintered material becomes part of the finished item, but the unsintered materials stay part of the supporting framework and must be removed during postprinting processing. The use of SLS in tissue engineering is widely known, and it has been successfully employed in a variety of other nonmedical industrial industries. Until now, it has not been employed in pharmaceutical applications, presumably because the high-energy input from the laser beam raises worries about the risk of drug and pharmaceutical excipient deterioration [56].

### 3.5. SLA

An SLA is a laser-based 3DP technique that utilizes photochemical reactions to generate layered prototypes, patterns, and models from UV-sensitive liquid resins (Figure 2(a)). The breadth of the cured layer depends on the energy of the UV laser beam to which the monomers and oligomers are subjected [40]. Fast, high-resolution, and scalable 3DP employing poly(N-isopropylacrylamide) for the creation of stimuli-responsive hydrogels may have diverse applications in drug delivery [57]. It was established that semisolid extrusion 3DP in conjunction with UV-LED curing at room temperature may be effectively used to make printable devices containing thermolabile pharmaceuticals [58].

## 4. 3DP-Based Pediatric Drug Formulations

The primary constraints and difficulties associated with pediatric medication formulations include the variability of the pediatric population, the absence of information regarding the safety and acceptability of formulations, the outcomes of pediatric clinical trials, and the scarcity of appropriate formulations tailored to the pediatric population. This can require the manipulation of adult medications, which can pose problems [59, 60]. Although oral delivery is the most well-known and prevalent method for pediatric patients, developing pleasant formulations is difficult. The created drugs should be easy to take and have a neutral taste while retaining safety, effectiveness, accessibility, and cost. Nonetheless, there is an increasing demand for the advancement of innovative technologies for young patients who mostly focus on formulation design and/or administration/dosing devices [61]. Such fresh expertise is 3DP, which encompasses a diverse variety of additive manufacturing procedures where things are developed using computer-aided design (CAD) software [12, 62]. The product is then handled using slicing software, which divides it into thin cross pieces. These are then printed one on top of the other to create solid layers that make up the 3D structure. The fundamental goal of the different 3DP methods is to progress and manufacture models and prototypes of the specified product in a timely and cheap manner. The recently allowed 3D-printed medication product named Spritam (levetiracetam), made utilizing the Zip dosage technique based on a PB-liquid 3DP technique, is an example of 3DP uses for medical items [63].

Several researchers are utilizing 3DP technologies to enhance oral dosage forms with variable drug release patterns that allow for either prolonged or rapid medication release [43, 64]. Polypill, a solid dosage form with several active components, comprising five different API compartments within a single shell that are independently regulated with two separate release profiles, was recently created using 3DP [65]. The research proposed an intriguing technique by combining FDM with SLA utilizing a nose-shaped structure loaded with an antiacne medication (salicylic acid) [66]. FDM has proved to be one of the best versatile 3DP processes because of the wide range of possible printable formulation components that the system could use. However, additional advancements in FDM extruders have permitted the printing of practically any thermoplastic material, such as polycarbonate and polyurethane [67].

The Food and Drug Administration (FDA) approved Aprecia Pharmaceuticals' Spritam, the first tablet to be 3D printed, in 2015. This levetiracetam-containing orodispersible tablet is made by spraying an adhesive solution over a PB, a process known as “drop-on-solid,” which produces a very porous tablet with a quick disintegration time [68]. The FDA's clearance of the first 3D-printed tablet brings the application and growth of 3DP expertise into the limelight [69]. As a result, the application of 3DP expertise in the pharmaceutical area and drug delivery systems has become a popular issue, especially in pediatric pharmaceuticals [70]. It is regarded as a life-saving approach that is useful for pediatric applications as a contemporary method for constructing individualized medicine delivery systems [62]. 3DP technology demonstrates the distinctive capability to manufacture oral tablets with customizable release characteristics for pediatric patients, such as immediate release, sustained release, delayed release, and target drug release systems [71]. Moreover, 3DP technology can produce orally dispersible tablets and orally dissolving tablets, which is mostly useful for successful compliance with young patients [48]. Furthermore, it is practicable to create and manufacture advanced oral dosage forms and particular devices intended for young patients, such as minicaplets and liquid capsules [72], inhalants, transdermal microneedles, and patches [73]. In the case of the pediatric population, a more appealing product might lead to increased patient compliance and treatment adherence. The recently developed 3DP expertise in the pharmaceutical business has substantially advanced the ease and variety of the creation of pediatric formulations [74].

**Figure 3:**
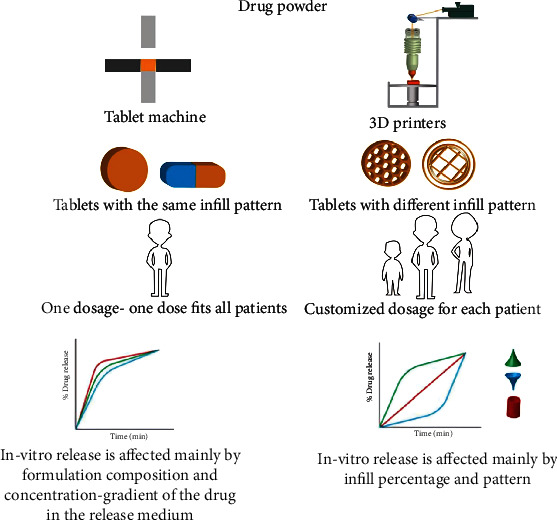
Illustrative sketch clarifying the cons and pros of 3DP and conventional techniques [73].

The 3DP technology can properly alter the advanced geometric forms of tablets and print varied colors and cartoon tablets to meet the children's specific tastes [75]. Significantly, not only can the unique form be made, but the required exact dosing scheme may also be satisfied. 3DP, as a new pharmaceutical manufacturing technology, brings production closer to the patient and can facilitate the delivery of precise doses, which is highly desirable in children's medicine where dose modification is required based on the patient's age, body mass, and clinical situations [68]. Tailoring the dose and medication distribution by making use of a flexible scheme and manufacturing dosage forms using 3DP will get the best out of what happens clinically in patients [76].  Figure 3 depicts an illustrative drawing that clarifies the benefits and drawbacks of 3DP over traditional compression approaches [73].

Pharmaceutical materials have recently made use of FDM, wherein FDM 3D printers are utilized to manufacture and extrude medication and polymer filaments. Specifically, formulations with different shapes and infill ratios have been created using FDM in order to alter the drug release profile [77]. As predicted, tablet weights increased with a rising infill percentage while remaining the same size, demonstrating great repeatability in physical dimensions as well as strong mechanical strength that is resistant to damage during handling. Furthermore, prolonged-release tablets of prednisolone were manufactured by utilizing loaded polyvinyl alcohol filaments [78]. The precision of dosage control was between 88.7% and 107%, whereas prednisolone was present in the amorphous form inside the matrix composed of polyvinyl alcohol and released from the 3D-printed tablet over 24 h. Other study focuses on the development of patient-specific immediate release tablets employing theophylline-loaded filaments as a combination in the presence of plasticizers generated via HME. A 3DP technology proved feasible to make caplet-shaped tablets with outstanding mechanical characteristics and an instantaneous in vitro release pattern [79]. The samples above demonstrate that drug dissolving rates may be altered by merely changing the printing patterns and structure without changing the polymer-drug characteristics.

The future 3DP technique boosts children's compliance and adherence to the prescribed medicine. Furthermore, the combined benefits of HME and 3DP, such as flavor masking and comfort of swallowing, contribute to dosage precision and improved palatability [16].

### 4.1. 3D-Printed Pediatric-Friendly Chocolate-Based Formulation

Cocoa-based products, such as chocolate and chocolate milk, have previously been employed as palatability enhancers due to their improved acceptance by the pediatric population [80]. Chocolate's health advantages have been claimed for many years, but only lately have certain of these claims been more precisely defined and investigated. For example, the antioxidant capabilities and favorable benefits in the cognitive performance of bioactive ingredients in cocoa and cocoa-related products have been carefully examined, and many health claims for cocoa polyphenols have been presented [81]. Meanwhile, preparation lines for dosage forms containing chocolate ought to consider potential food-drug interactions caused by chocolate's tyramine and caffeine content, circumventing certain medication classes, including monoamine oxidase inhibitors, some bronchodilators (e.g., theophylline), and antibiotics (e.g., ciprofloxacin).

**Figure 4:**
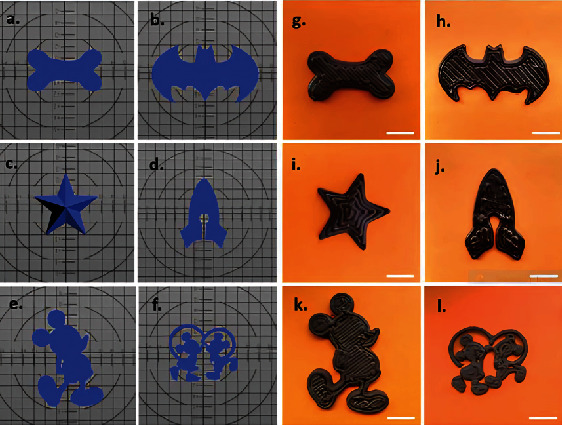
(a–f) Schematic picture. (g–l) .stl files. 3D-printed chocolate-based dosage forms [75].

Karavasili et al. created a combination of bitter chocolate and corn syrup at various ratios (1:0.5, 1:0.6, 1:0.7, 1:0.8, 1:0.9, 1:1, and 1:1.2) utilizing paracetamol (PCT) and ibuprofen (IBU) as model drugs [75]. Masterpieces with low syrup proportions (≤1:0.7) and viscosity at shear rate = 0.1. The ratio of syrup to chocolate was adjusted further to enhance pharmacological loading, with a higher ratio (chocolate:syrup: 1.2) leading to difficult-to-handle sticky ink compositions. As a result, a ratio of 1:1 (*w*/*w*) chocolate to syrup was chosen for future investigation. This combination allowed for seamless extrusion of self-standing multilayered prints ranging from simple shapes such as a star to cartoon figures (Figure 4), with the printing procedure taking only 8 min [75]. The 3D-printed chocolate dose forms ranged in weight and dimensions (*L* × *W* × *H*) from 5.2 g and 59.1 × 33.1 × 3 mm (Figure 4(g)) to 17.9 g and 61.8 × 84.1 × 6 mm (Figure 4(k)). One of the primary benefits of 3DP expertise is the skill to personalize medicine dosage by readily manipulating both the weight and size parameters of the dosage forms, allowing for dose modification based on the pathophysiology of each unique patient. The drug loading was determined to be 100.84% (±0.67%) for PCT inks and 99.5% (±1.49%) for 3D-printed formulations and 100.3% (±1.6%) and 98.2% (±4.7%) for IBU inks and 3D-printed formulations, respectively. The agreement comparing the real and expected drug content for both active substances suggests drug content consistency in the produced formulations, which is extremely desired to permit control over dose accuracy in the final dosage forms [75]. The pediatric population is more sensitive to this problem since adult doses are administered using the standard dosage-splitting approach.

### 4.2. 3D-Printed Colorful Cartoon Model Printlets

**Figure 5:**
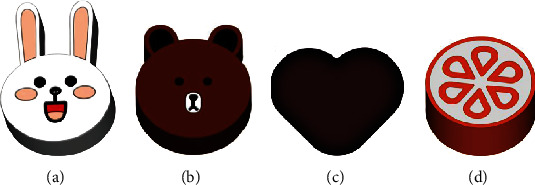
Schematic illustration of the colorful cartoon models. This can be chanted according to the preferences of children to improve their medication compliance. (a) Rabbit, (b) bear, (c) heart, and (d) candy [80].

Using the CAD program 3D Sprint (3D systems, United States), vibrant cartoon models with kid-friendly characteristics like a heart, sweets, and cartoons were made (Figure 5), making them easier for youngsters to carry about. Tablet strengths inside spatial configurations (such as lattice structures, solid systems, hollow frameworks, and hollow arrangements with internal support) and as a function of tablet size can be tailored to achieve specific dosage and release characteristics [80]. Furthermore, the tablets' form and color may be altered according to children's preferences [80].

**Figure 6:**
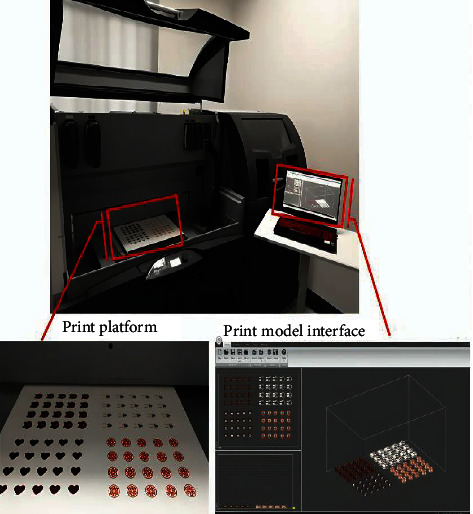
Schematic diagram of the process of colorful cartoon tablets using a binder jet 3D printer [80].

The researcher printed with a binder jet 3D printer (Projet CJP 660 Pro, 3D Systems, United States). As illustrated in Figure 6, the created model file was uploaded to the 3D printer's software, which then sliced the model and transmitted the slices to the printer [80]. The platform was covered with thin layers of powder mixture (100 *μ*m each). Five hot-bubble printing heads (HP11, Japan) on the print carriage were used to accurately deposit clear or colored inks with specific compositions as they went over each layer. These inks were placed in a cartridge with a 10 *μ*m filter. Every printing head contains 304 nozzles, and each nozzle's single droplet is 18 pl in size. The ink only hardened the powder in the planned model's cross-section, leaving the rest for support. The print resolution was 600 × 540 dots/in. (DPI), with a maximum vertical build pace of 28 mm/h. After printing, the tablets were dried at 400°C for at least 15 h to eliminate organic solvents and excess moisture, and the support powder was recovered for reuse using an integrated vacuum system. The tablets were then cleaned using an airbrush to eliminate superfluous powder [80, 82].

**Figure 7:**
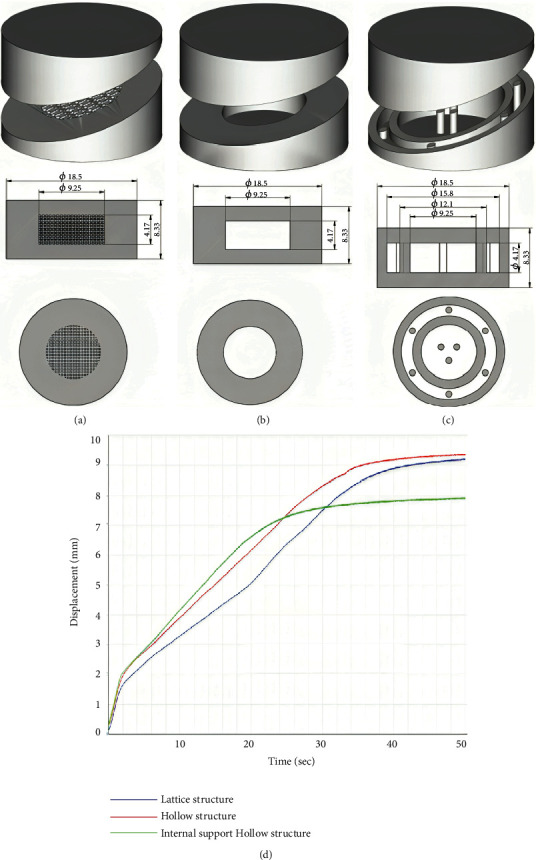
Schematic image of the different internal spatial structure models and disintegration curve. This is designed to achieve faster disintegration and maintain the mechanical strength of the tablets. (a) Lattice structure; (b) hollow structure; (c) hollow structure with internal support; (d) disintegration curve of the tablets with different internal spatial structures determined by a texture analyzer at a constant pressure [80].

Three types of internal spatial structure models were constructed, as shown in Figures 7(a), 7(b), and 7(c), to accelerate drug release: lattice structure, hollow structure, and hollow structure with internal support [80]. The lattice structure and hollow structure models have a shell thickness equal to one-fourth of the tablet size. Based on the hollow structure concept, the nine columns in the shell and the hollow section are consistently distributed throughout the structure to accomplish rapid disintegration while maintaining the tablets' mechanical qualities. According to the findings, the average hardness was only 13 N, suggesting that the mechanical qualities of the hollow structure with the internal support tablet were inadequate, and the tablet failed the friability test. The hollow and lattice-structured tablets had an average hardness of 61 and 59 N, respectively, and the tablet remained intact following the friability test. In expressions of the texture analyzer's disintegration curve at persistent pressure, shown in Figure 7(d), the hollow structure tablet disintegrated faster and had mechanical properties similar to the lattice structure, so it was chosen as the tablet model [80, 83].

**Figure 8:**
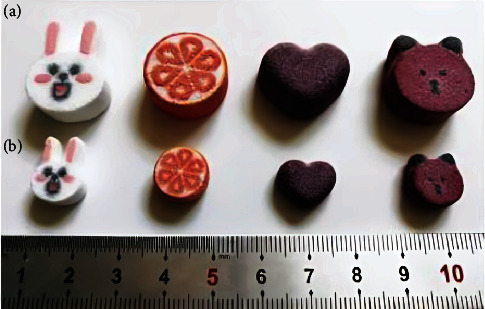
3D-printed cartoon tablets with different strengths. (a) 1000 mg strength; (b) 250 mg strength [80].

3D Sprint created cartoon tablets with hollow structures in various strengths (1000 and 250 mg) and looks. To reduce the consumption of pigment, color printing inks were used solely on the tablet's exterior layer, while clear printing ink was utilized on the tablet's inner layer, with the exemption of the hollow area. As demonstrated in Figure 8, cartoon tablets were manufactured with great precision and repeatability without any faults, which might increase children's medicine compliance [80, 84].

**Figure 9:**
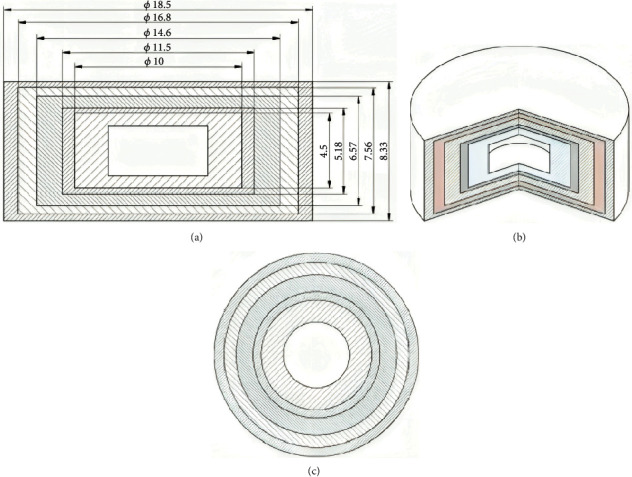
Schematic diagram of different size models for adjusting the drug dose. (a) Side view dimension profile; (b) three-dimensional profile; (c) top view [77].

Through the scheme of model size, 3DP technology enables the flexible modification of medicine dosage for tailored drug delivery. However, in order to accomplish the need for precision printing for tablets of various model sizes, the printing ink, powder, and printing parameters must all work together. The study found that 3D Sprint software was used to produce tablet forms of various sizes, with theoretical strengths of 160, 250, 500, 750, and 1000 mg. A schematic design of the relevant dose model is presented in Figures 9(a), 9(b), and 9(c). Tablets have a corresponding diameter and height. Hollow model tablets of various sizes were printed using predetermined printing ink and powder, and the weight and content (used to compute strength) of each tablet were determined [80, 85].

### 4.3. 3D-Printed Solid Dispersions

HME is a well-known pharmaceutical processing method that has been used effectively for the past two decades to enhance the solubility of water-insoluble medications and conceal the taste of bitter APIs in pediatric applications by generating solid dispersions. In the study, HME was the predominant way of creating printing filaments. Which were then connected with a 3D printer. Recently, HME has become the major technique for creating pharmaceutical filaments that can then be placed into a 3D printer. Extrusion or soaking introduces the API [86]. Indomethacin (IND) was utilized as a model API in research that produced extruded solid dispersions with enhanced solubility, while hypromellose acetate succinate (HPMCAS) is a suitable thermoplastic polymer for the manufacturing of drug-laden strands. The study's goal was to create taste-masked IND/HPMCAS solid dispersions with improved drug solubility in the form of strands/filaments that could be fed into a 3D printer to make pediatric chewable tablets. According to previous research, children's capacity to swallow solid dose forms is a key concern; hence, chewable tablets make an effective pediatric dosage form. They can be administered to youngsters aged 2 and up if swallowing or disintegration is aided by the patient [87].

The researcher was motivated by the Starmix flavor gummy sweets (HARIBO Ltd.) to emerge as “candy-like” formulations. Imitating these popular confectionaries, the 3D-printed chewable tablets seek to increase pediatric population compliance and palatability, which is the major issue for caregivers and health professionals to deliver proper healthcare for these sections of patients. Premixed drug, polymer, and plasticizer mixtures were introduced into the extruder to make drug-loaded filaments appropriate for 3DP. One of the best important features is the filament quality, which should be flexible enough to avoid breakage during printing. To improve extrusion processing and strand flexibility, polyethylene glycol (PEG) was used as a plasticizer. Various polymer/plasticizer ratios tested revealed that 20% PEG provided the best filament quality. To meet the 3D printer specifications, the filament diameter was adjusted to between 2.7 and 2.9 mm. Because of the HPMCAS/PEG swelling by the end of the extrusion process, a 1 mm die was selected to process the formulations [16].

**Figure 10:**
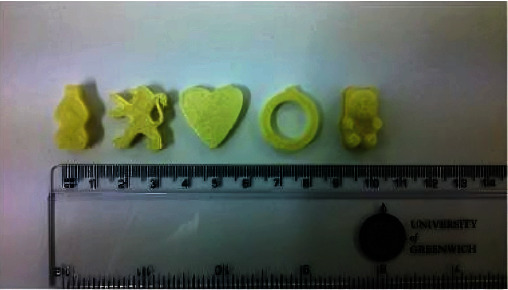
Photographic image of the 3D-printed medicines loaded with IND as model substance [16]. IND: indomethacin.

In Figure 10, various Starmix IND-loaded structures were printed in a variety of designs using Solid Works. The process was automated with a 5% infill ratio, a printing speed of 25 mm/s, and a layer thickness of 160 *μ*m to print doses of 25 mg IND per assembly. Each Starmix tablet took 5 min to print [16].

### 4.4. 3D-Printed of Minitablets

Children can more easily ingest minitablets (diameter < 5 mm) than bigger tablets or capsules. One way to modify the dosage is to alter the quantity of minitablets given. Minitablets may be precisely fabricated with perfect control over shape and size with the help of 3D printing. Drug release can be improved by adding channels to minitablets to increase their surface area [88–90].

The study found that drug-loaded filaments can be successfully extruded at temperatures of 1700 C for hypromellose (HPMC) filaments and 1400 C for hyprolose (HPC) filaments. These filaments showed flexibility for further processing and a homogenous diameter of around 2.8 mm, suitable for 3DP. They were used to create tiny tablets with diameters of 1.5, 2.0, 3.0, and 4.0 mm. Each batch of microtablets was produced in four distinct sizes. The tablet shape was created with Free CAD 0.18 and saved as a SLA file in Cura. The small tablets were created using a FDM printer, the Ultimaker UM 3 [88]. SLS 3DP has also been successfully used to manufacture miniprintlets with diameters of 1 and 2 mm. In the literature on SLS for miniprintlet formulation, PCT was used as the model medication, while ethyl cellulose was used as the primary polymer matrix. The laser scanning speed of 50 mm/s was chosen because it was discovered to provide adequate energy for the efficient bonding of consecutive printing layers while preserving the appropriate form and dimensions of the miniprintlets [91]. One batch of 100 1 mm miniprintlets took around 2 min to print, whereas a batch of 2 mm miniprintlets took approximately 2 min and 40 s [38].

Dual miniprintlets for multidrug therapy were also developed, including PCT and IBU in distinct layers. Similar to single miniprintlets, dual miniprintlets come in two sizes: 1 and 2 mm. Two distinct arrangements of the dual miniprintlets were created: one medication was distributed in Kollicoat IR, a polyvinyl alcohol/PEG graft copolymer with fast-release properties, while the other medication was distributed in ethyl cellulose. Printing 100 double miniprintlets of 1 mm took around 2 min and 30 s, whereas printing 2 mm miniprintlets took about 3 min and 40 s [92]. The duration is significantly longer than for the single miniprintlets because the powders were manually applied, which needed additional surface heating [38].

### 4.5. 3D-Printed Gummy Formulations

Together with the active medicinal component, the gummy formulations are usually made of gelatin, HPMC, reduced syrup, and water. The viscosity and printability of the formulation are impacted by the gelatin and HPMC [93]. Custom gummy medicine formulations that are suited to the unique requirements of pediatric patients can be made possible via 3D printing. Gummies' delicious flavor and smooth texture make them a popular dose form for kids [94].

**Figure 11:**
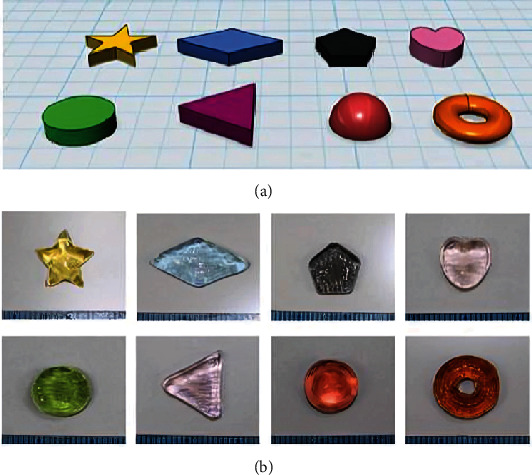
Fabrication of gummy formulation with various shapes and colors. (a) Design; (b) 3DP. (a) Design of objects with various shapes and colors. (b) 3D-printed gummy formulations with various shapes and colors [93].

The various forms of the gummy formulations (square, star, diamond, pentagon, heart, cylinder, triangle, hemisphere, and doughnut) were created with 123D Design (Autodesk Inc., San Rafael, CA, United States). The printing settings were determined using the Slic3r Slicer software (GNU General Public License; printing velocity, 10 mm/s; travel velocity, 80 mm/s; layer height, 0.3 mm; vertical shell, 1; fill density, 100%). To eliminate bubbles, each finished medication formulation was placed into a 3D printer syringe and centrifuged at 500 × g for 5 min. The filled syringe with 27G nozzle was inserted in the 3D bioprinter (INKREDIBLE; CELLINK; Gothenburg, Sweden), and the sample was 3D printed at room temperature (25°C) with the 3DP software (Repetier-Host Software; Hot-World GmbH & Co. KG, Willich, Germany) before being dried and solidified for 24 h. Incorporating an adequate amount of gelatin and HPMC permitted the manufacturing of gummy formulations with the correct shape. The printability (look) of a gummy formulation was strongly tied to its viscosity [93]. As seen in Figure 11, gummy medication formulations were extruded into a variety of forms and colors as a step toward customized medicine. Children have color and form preferences, which may emerge during socialization or cognitive gender development [93]. 3D printers are excellent for producing medications in a variety of forms and colors for pediatric patients. The study disclosed an FDM-type 3D printer for producing tablets of various forms and capsules of various colors, as well as an investigation of patient acceptance with adult volunteers. 3DP of pharmaceuticals is adaptable and suitable for pediatric patients. Their group conducted a crossover trial on chewable printlets in various colors and flavors against juvenile patients (3–16 years old) with maple syrup urine illness [95]. Although conventional methods may be used to create gummy formulations, 3D printers can produce items with a variety of complicated forms on demand in a therapeutic context.

The researchers included the model medication lamotrigine in the formulations (less than 1 mg in each gummy tablet). Gummy formulations with higher doses are planned to be prepared in the future. An increase in lamotrigine, a weakly water-soluble medication, may have an effect on the formulation's viscosity design as the solid concentration increases. Tagami et al. determined the quantity of reduced syrup in the medicine formulation. The viscosity of the medicine formulation can be altered, and the bitterness of the gummy formulation may be disguised by adding more reduced syrup [93].

### 4.6. 3D-Printed Dose-Control Platform

A versatile manufacturing platform provided by 3D printing technology may be able to meet the demand for customized dosage forms, including accurate layer-wise dose modifications for pediatric usage [68]. In this effort, the cylindrical technique was chosen as the fundamental digital model for a series of produced tablets. The computer-generated models were created using Autodesk CAD 2010 and stored as SLA files (.stl) [96]. The models were then imported into the 3D printer's software control system and converted to G-code for printing. The printing model that simultaneously regulates the dimensions of the tablets, both diameter and height (*D*, *H*), offers a much more effective and accurate regime for manufacturing tablets with the expected dose. As a result, a succession of 3D-printed cylindrical tablets of increasing volumes were produced [97].

The active medicines and excipients were run through a 150-mesh filter to regulate particle sizes of <90 *μ*m. To guarantee homogeneity, 1 km of mixed powder was placed in a plastic bag and stirred for 20 min. The powder was then released into the 3D printer's supply tank. The volumetric ratios of the printing ink were 100:20:5:3 for acetone, ethanol, deionized water, and glycerin, which were evenly blended and then put into the ink container (Hp 816) [98].

The computer program constructs three-dimensional objects by repeating the printing instructions. The printing nozzle moved in the *x*-*y* axis direction to position the powder in a specified area, after which the printing ink was sprayed once on the initial layer. Following that, the printing platform slid 0.10 mm downward along the *z*-axis. The processes outlined above were repeated until the desired size was obtained. The overall printing time for each tablet was roughly 20 min. After printing, the printed tablets were allowed to stand and firm for 30 min at room temperature before the support materials and powders on their surfaces were removed. The printed tablets had been dried in a blast oven at 40°C for 24 h [98].

Throughout the printing process, the printing settings are critical in ensuring the dimensional stability of the 3D-printed tablets. Therefore, the important parameters were optimized by the single-factor screening method and set as follows: the spraying rate (4 nL∗12 kz), the printing temperature (room temperature), the layer height (0.10 mm), the interval time (7) between printed layers (0.25 min), and the number of tablets printed at the same time [99].

### 4.7. 3D-Printed Oral Films

Swallowing films (OFs) are a relatively recent dosage type that delivers medications through the mouth cavity or oromucosal route. They have recently gained popularity for usage in youngsters and people with physiological or psychologically caused dysphagia [100]. Pullulan (PUL) has recently been shown to be suitable for OFs due to its excellent film-forming characteristics [101]. It possesses significant mechanical strength and water-soluble properties, in addition to being odorless and tasteless [102].

A study was conducted to develop hybrid film structures using PAMs while avoiding the problem of merging immiscible polymers. Three formulations were first printed: the clean polymer solution (P0/H0), 3% *w*/*w* caffeine (P1/H1), and 10% *w*/*w* caffeine (P2/H2). Excessive printing forces were required for the latter, which pushed P2 to the limits of the PAM printer. As a result, two new formulations were created, with water content raised by 3% and 6% *w*/*w* to lower viscosity and, consequently, printing pressure. This section begins with the outcomes of the characterization of the feedstock and then moves on to the results for the final product. For the feedstock (i.e., the solutions), multiple rheological characterization methods were used, as rheology is a valuable indication of a material's printing performance [103]. The result (the films) was appraised in terms of chemical structure, shape, mechanical qualities, and disintegration. PAM was used effectively to manufacture caffeine-loaded PUL, HPMC, and PUL-HPMC composite films. The printing variables were discovered to change according to the formulation. P0 and H0 were printable at 65 kPa, whereas P1 and H1 were printable at 80 kPa, representing a 23% increase in pressure. P2 and H2 needed 196 and 140 kPa, which corresponded to 145% and 75%, respectively, a significant increase compared to prior formulations. The use of water lowered the necessary printing pressures to 60 and 40 kPa for P3/H3 and P4/H4, respectively. Thus, raising the water level from 60% to 66% *w*/*w* for films containing 10% w/w caffeine and lowering the biopolymer content from 23% to 17% *w*/*w* had a significant influence on extrusion pressure. Despite increased water content, the films maintained their 30 × 10 mm rectangular form. The thicknesses of the films measured using a Vernier caliper and scanning electron microscopy (SEM) varied from 100 to 150 *μ*m [102].

The study's findings show that PAM is a simple, cost-effective, and quick option for hybrid film manufacturing. The main discovery of this effort was that PUL might be combined with HPMC to generate a viable film when treated using PAM, with a rise in PUL's mean tensile characteristics. Furthermore, it was discovered that the printing orientation of PUL-HPMC affected its tensile qualities. Thus, in addition to the two polymers' intrinsic qualities, the printing orientation may be used to adjust the mechanical properties. 3DP, as previously said, is capable of producing complicated structures. Given that juvenile kids are frequently the intended beneficiaries of OFs, the ability to construct various and appealing forms can make the drug more appealing. The study also compares it to other methods, like injection molding and extrusion, which require expensive molds or dies to produce diverse forms. Furthermore, typical production procedures have limited capability for individualized, on-demand distribution when demographics such as pediatrics require significantly variable doses [30]. However, current clinical studies show that 3DP can achieve this aim [95]. As a result, 3DP has the ability to address a clinical requirement for on-demand OF creation. Previous work has employed FDM, a popular 3DP technique, to produce films. The study claimed film thicknesses of 197 *μ*m, which are more than the 100 *μ*m created here [104]. Furthermore, PAM does not necessitate the use of expensive equipment to generate the feedstock, whereas FDM does require hot melt extruders. PAM has the benefit over most 3DP technologies in that it can process a varied range of polymers, whereas FDM is confined to thermoplastics. The film thicknesses were similar to those described by IJP, a 3DP method extensively utilized for OFs, even though IJP may attain lower thicknesses [105]. However, IJP has limitations in terms of drug loading, whereas PAM can accomplish stacking of up to 80% *w*/*w*. More effort will be required to fully exploit the promise of PAM for the manufacturing of OFs [102].

### 4.8. 3D-Printed Novel Bespoke Soft Dosage Form

A 3DP using an M2 MakerGear FDM 3D printer and ABS filaments (MakerGear LLC) and simplified 3D software produced the template design. A Lego-inspired design was chosen to demonstrate that e-3DP could generate a complicated shape and a form recognizable to youngsters. A Lego-like template was created using PLA filament (MakerBot). The design that was used for printing the paste with active ingredients was created using Autodesk 3ds Max Design 2018 (Autodesk Inc., San Rafael, CA, United States). Simplify 3D software and a modified MakerBot Replicator Experimental 2X were used to print the paste containing the active substance in the proper design during the experiment. The implanted printing arrangement was 20 × 29.52 × 0.45 mm and was tailored to fit into a gelatin-based Lego-like block (40 × 25 × 15 mm). For 25%, 50%, and 75% printing arrangements, the schemes had equal *X* and *Z* extents; however, the *y*-axis was 8.1, 15, or 22.5 mm. Printing the whole pattern (100%) yielded dosages of roughly 107 and 115 mg for IBU and PCT, respectively. To illustrate the system's ability to print lesser and greater dosages, the printing pattern was printed at 25%, 50%, or 75% of the whole design for lower doses and twice (200%) or three times (300%) for bigger doses. All printing patterns were produced using identical Lego-like templates, as described above [47]. The process may also be one-step embedded 3DP or multistep embedded 3DP.

**Figure 12:**
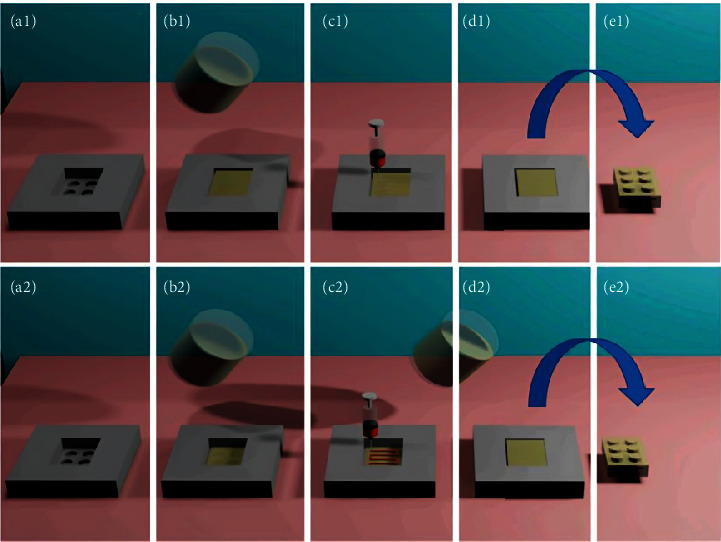
Figure depicting the single-stage and the two stages of the e-3DP process [106]. (i) In a single printing step, a gelatin-based matrix liquid is poured into a (a1) 3D-printed template at (b1) 700°C, and the (c1) drug paste is then extruded into the liquefied matrix. After bringing the template down to (d1) room temperature, an (e1) oral dosage form is taken out of it. (ii) Two-stage printing: (a2) pour 70°C of gelatin-based matrix liquid into the template, (b2) let it cool to room temperature, (c2) extrude the drug paste, (d2) pour a second layer of gelatin matrix over the template surface, and (e2) extract the dosage form [106].

To implant a drug solution design into warm gelatin, heat the medium to 70°C, cast it into a Lego form template, and lay it on a 75°C 3D printer printing plate. The Simplify 3D software was used to design parameters such as locations, dimensions, printing speed, and extrusion multiplier (Figures 12(a1), 12(b1), 12(c1), 12(d1), and 12(e1)). To print the embedded layer(s), heat the building platform to 75°C and pour gelatin into the template. To enable the needle tip to start extrusion at a height of 4 mm from the bottom of the design, the G-code was altered. G16 was the needle size, and 10.0 was the extrusion multiplier. The following needle speeds were tested for printing patterns: millimeters per minute: 50, 55, 60, or 65 [106].

#### 4.8.1. Multistep Embedded 3DP

The drug was incorporated into the structure in three steps: (i) creating a liquid gelatin solution (6 mL) inside the template and allowing it to cool to harden, (ii) printing the drug paste (embedded phase) on the outermost surface of the semisolid gelatin, and (iii) covering the drug paste with another portion (5 mL) of liquid gelatin. To allow for the printing of two model medicines at differing specific levels, 4 mL of anchoring phase was cast first, followed by PCT printing, and finally 3 mL of embedding phase. After printing the IBU layer, a final coating of gelatin (4 mL) was added.

**Figure 13:**
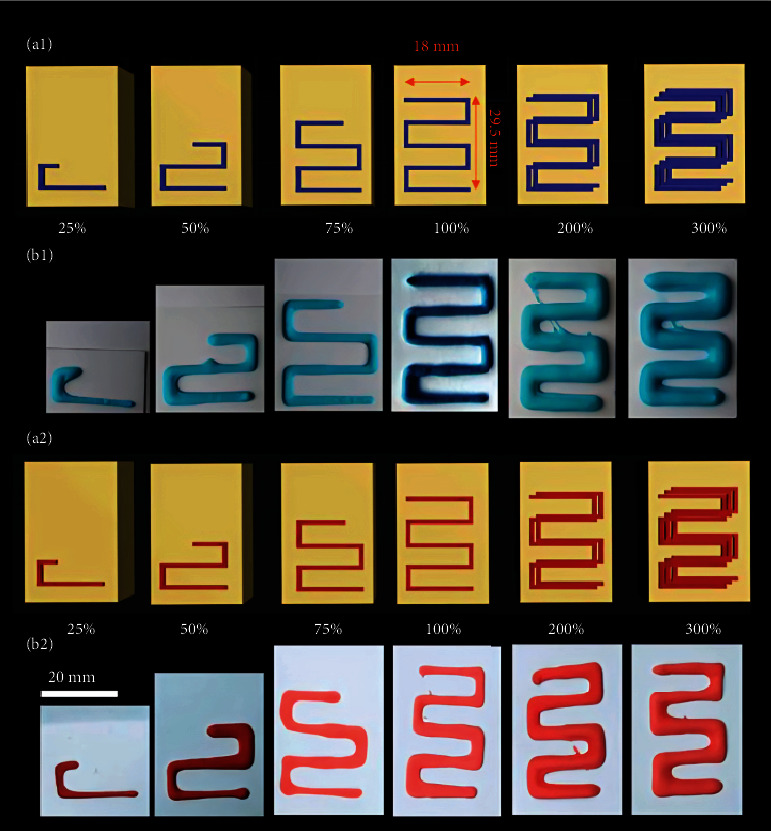
(a1, a2) Rendered and (b1, b2) photograph images of printing patterns for PCT and IBU [47]. PCT: paracetamol; IBU: ibuprofen.

The printing configuration was cut or reproduced at varied proportions to demonstrate the system's capacity to provide a variety of dosages (Figure 13). For example, to obtain lesser dosages, printing patterns were decreased to 25%, 50%, or 75% to cover PCT and IBU dose ranges of 16–77 mg and 12–76 mg, respectively. To produce greater dosages, the printing configuration was repeated twice or three times along the same printing route, followed by a 0.5 mm height increase after each layer. Figure 6 displays rendered pictures and photos of printed patterns made using this method for PCT and IBU. The study also found that both model medications had strong linearity (*R*^2^ = 0.9804 [PCT] and 0.9976 [IBU]) between the percentage of printed configurations and the attained dosages [47].

The e-3DP technology offers several advantages in chewable goods development, including the ability to encapsulate medication paste in a gelatin matrix, allowing for high levels of individualization and intricate dosage schedules. Its modularity allows for the incorporation of tailored pharmaceuticals and doses in a single chewable form, enabling more complex dosage schedules. Age-tailored dosage forms can accept multiple medications in fewer doses, making them suitable for therapeutic situations where integrated treatment is needed. The technology also allows for quick medication combinations in varied ratios, benefiting families by eliminating the need for multiple drug administrations [107].

The study demonstrates the potential of e-3DP for creating chewable, soft dose forms suitable for various age groups, especially youngsters. The soft texture and sweet flavor could benefit those with swallowing difficulties or palatability issues. The technique allows for the independent development of shell and core components without altering matrix composition [47].

### 4.9. Current Clinical Trials on 3DP-Based Pediatric Drug Formulation

Currently, the FDA has authorized only one 3D-printed medication for human use, which is noteworthy when considering pediatric formulations: levetiracetam or Spritam. Spritam is an orodispersible tablet that was approved by the FDA in 2015 and is used to treat epilepsy. This drug makes use of ZipDose technology from Aprecia Pharmaceuticals, which enables high-dose formulations that quickly dissolve with only a sip of water. This feature makes it simpler for kids who have trouble swallowing regular pills [19, 108].

Although the primary indication for Spritam is epilepsy, its approval highlights the potential of 3D printing technology to create dosage forms that are suitable for younger patients. This achievement has sparked additional investigation and the creation of additional 3D-printed drugs tailored to the unique requirements of pediatric patients. The following remarkable products are presently undergoing clinical studies for pediatric medication formulations made via 3D printing.

#### 4.9.1. Spanish Hospital Trial

A hospital in Spain is conducting a clinical trial to evaluate the efficacy, tolerability, and acceptance of a 3D-printed drug specifically designed for pediatric patients. This is the first of its kind in Europe for pediatric applications and is a collaboration between Vall d'Hebron University Hospital, the University of Santiago de Compostela, and FabRx. The 3D printer produces semisolid and chewable medicines personalized to each child based on their weight and clinical characteristics. This new method of administering medication is more convenient and avoids dosing errors compared to traditional syrups. The trial will also assess the efficacy of the new formulation and its impact on acceptability and the experience of minors and their families. The 3D-printed medications can be customized based on each child's preference and do not need refrigeration, increasing safety and ease of transport [19].

#### 4.9.2. FabRx M3DIMAKER

Clinical trials have employed FabRx's GMP-ready 3D printer, M3DIMAKER, to create customized pharmaceuticals. Interestingly, chewable “printlets” for kids with maple syrup urine disease (MSUD) have been made using it. But because there are currently no commercially available oral therapies for MSUD, clinicians must manually manufacture formulations (by weighing powder and manually filling capsules). In order to overcome these obstacles, the University Clinical Hospital in Santiago de Compostela (Spain) integrated FabRx's 3D printer within its pharmacy department. This breakthrough made it possible to produce appealing chewable printlets with isoleucine in a range of doses, flavors, and colors. These were tested for patient acceptance and treatment control [95, 109].

In comparison to the usual medication (capsules), the researchers discovered that 3D printing allowed for finer control over target blood concentrations. They also discovered that all patients approved of the flavors and colors of the 3D-printed dose forms. In addition, FabRx created a cutting-edge new 3D printing technology in 2019 for the manufacturing of drugs. Direct powder extrusion (DPE), a revolutionary printing technology, makes it possible to build drug products directly from powdered ingredients in a single step, eliminating the time-consuming procedures often needed to produce 3D printer filament feedstock, which is utilized in FDM printing. This technique offers potential for use in preclinical research and early-stage clinical trials since it allows for customized and adjustable dosage with short development timelines [110].

#### 4.9.3. Triastek

Triastek received FDA IND approval for its 3D-printed drug T19, designed to treat rheumatoid arthritis using melt extrusion deposition (MED) technology. This drug is undergoing clinical trials and is expected to file a new drug application (NDA) in the near future. The technology allows precise control over drug release profiles, which can be particularly beneficial for pediatric patients requiring tailored dosing [111].

#### 4.9.4. Merck's Exploratory Studies

Merck is testing various 3D printing technologies, including powder jetting and material extrusion, to produce oral solid dosage forms. They plan to supply these formulations for clinical trials and aim to develop GMP solutions for market introduction within the next few years [112]. These trials highlight the potential of 3D printing technology to revolutionize pediatric drug formulation by providing customized, patient-specific medications that improve adherence and therapeutic outcomes.

## 5. Challenge and Opportunity of 3DP-Based Pediatric Drug Formulation

The field of pediatric 3D printing regulations is currently developing. The procedure becomes more difficult when one must ensure adherence to laws governing drugs and medical devices. Standards for safety and efficacy must be fulfilled, which might call for a lot of testing and validation. The safety of the materials used in 3DP has to be carefully assessed, especially in formulations meant for young patients. Certain materials require stringent screening and testing methods because they may contain poisons or allergies that might damage children [113].

It can be difficult to get 3D-printed formulations for pediatric usage to the appropriate degree of precision and accuracy. The quality and uniformity of the finished product can be impacted by changes in printing factors, such as layer thickness and printing speed. It can be challenging to increase 3D-printed pediatric formulation manufacturing to meet demand. Large-scale production may be hampered by the present limits of 3D printing technology, such as sluggish printing rates and small build volumes. 3DP can be costly, particularly if unique formulations are needed for every pediatric patient. Materials, equipment, and professional labor can be prohibitively expensive, especially for healthcare providers with limited funding [111].

Not every medication formulation lends itself to 3D printing. The variety of pharmaceuticals that can be generated with this technique is limited since some drugs might not work well with the printing process or might break down while printing. The pediatric investigation plan (PIP), mandated by the pediatric regulation, is a particular regulatory need for pediatric pharmaceutical formulations that are 3D printed. A PIP is a development plan designed to make sure that the information required to support the approval of a medication for children is gathered through research conducted on children. Despite the need for a PIP for medications meant for pediatric use, there is still a significant market for pediatric-focused pharmacological formulations [114].

There are moral questions regarding patient autonomy, informed consent, and equitable access to healthcare raised by the use of 3D printing in pediatrics. Patients and their guardians must give their informed consent because 3D-printed compositions can contain cutting-edge excipients or manufacturing techniques. Initially, the technology might only be available in a few locations or healthcare facilities, which could disadvantage underprivileged communities. Patient confidentiality and data security are also crucial. Because several parties are involved in the manufacturing process, it is crucial to establish clear lines of accountability and liability for the safety and effectiveness of pediatric medications manufactured via 3D printing. Creating strong regulatory frameworks is essential to guarantee the effectiveness, safety, and quality of pediatric formulations made by 3D printing [113].

The ethical issues surrounding 3D-printed pediatric medications have been addressed through proposed guidelines. These include Informed Consent Guidelines for comprehensive disclosure of novel formulations, Equitable Access Guidelines for accessibility to all children, Privacy and Data Protection Guidelines for personal and medical information, Accountability and Liability Guidelines for stakeholder roles, and Regulatory Guidelines for approval and postmarket surveillance. Collaboration between industry, academia, and regulatory bodies is encouraged to address unique challenges and ensure flexible regulatory frameworks that maintain high standards of quality and safety [111]. So in order to overcome these challenges, cooperation between medical professionals, government regulators, academic institutions, and business partners will be needed to create standardized procedures, strengthen material safety, advance printing technology, and guarantee accessibility and affordability for underprivileged children [115].

The possibility of an unintentional overdose is one issue with pediatric drugs, particularly when they are packaged as delectable candy or flavored goods. Because of the delicious flavor, kids can be tempted to take more than the recommended amount. This concern emphasizes how crucial it is to use cautious dosage and packaging, as well as to provide caretakers with clear instructions. Certain children may be at risk for allergic reactions due to color additives used in 3D-printed pediatric pharmaceuticals. For example, allergic reactions have been linked to synthetic colorants. To reduce the possibility of negative responses, careful color additive selection and thorough testing for allergenic potential are crucial [113].

3D printing can be used to customize medication for individual patients, reducing the risk of medication mistakes. This technology allows healthcare professionals to accurately regulate the dose and composition of pharmaceuticals, ensuring the correct substance is supplied in the right formulation. This is particularly beneficial for young patients who may require lesser dosages or customized formulations not commercially available [115]. 3D printing enables pharmacists to design personalized dosing for young patients, reducing the risk of overdosing or underdosing. This improves medication safety and effectiveness. 3D printing can produce various dose forms, including liquids, solids, and transdermal patches, based on the patient's age, health, and preferences. This allows pharmacists to choose the best route of administration for optimal therapeutic results. Additionally, 3D printing can expedite pediatric pharmaceutical turnaround times, ensuring prompt access for those in urgent need [116].

By enabling on-demand pharmaceutical manufacture, 3D printing has the potential to completely transform the pharmacy logistic supply chain. This can save waste, enhance inventory control, and eliminate the need for significant stocks of medications that have already been created. Furthermore, 3D printing can improve pharmacy operations' flexibility by enabling quick adjustments to shifting patient demands and new developments in healthcare [117].

If suitable steps are taken to address possible risks and limitations, using 3D printing in pediatric drug formulation provides the potential to improve medicine safety, effectiveness, and patient outcomes.

## 6. Future Perspectives

The future of 3D printing in pediatric drug formulation holds great promise, with key areas for continuous innovation and research to advance the field. Developing novel bioinks and smart polymers is crucial for enabling more sophisticated 3D-printed pediatric formulations. Advancements in CAD software and hardware are essential for translating patient data into printable dosage forms. Artificial intelligence (AI) has immense potential to enable truly personalized pediatric medicines through 3D printing [77].

To translate these innovations into commercial pediatric products, continued research is needed to establish regulatory guidelines for the quality, safety, and efficacy of 3D-printed medicines. Clinical trials should be conducted to validate the performance and patient acceptability of 3D-printed pediatric formulations and to demonstrate the long-term stability and shelf-life of 3D-printed dosage forms [113].

## 7. Summary

3DP technology has revolutionized the development of pediatric drug formulations, allowing for personalized dosage forms and precise dose adjustments. This technology also allows for complex drug delivery systems with controlled release kinetics, particularly beneficial for pediatric populations. 3D printing has shown promise in addressing common pediatric formulation challenges, such as taste masking and enhancing the solubility of poorly water-soluble compounds. While the FDA approval of Spritam in 2015 marked a milestone, further research and regulatory guidance are needed to fully realize the potential of 3D printing in pediatric drug development. The integration of advanced technologies like AI and smart materials will drive innovation in this field.

## Figures and Tables

**Table 1:** Latest innovations in dosage form geometry using 3DP.

**Description**	**Image**	**Reference**
3D-printed tablets of cylindrical and geometric lattice shapes fabricated using SLS 3DP	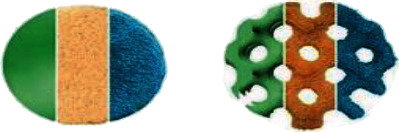	[36]
3D-printed multicompartment capsular devices for oral medication administration with two pulses	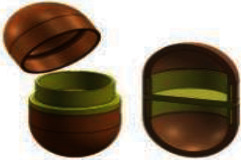	[37]
3D-printed pellets containing paracetamol and caffeine (1 and 2 mm) using SLS	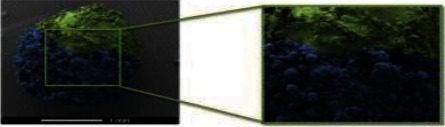	[38]
6-layer polypill in cylindrical and ring-shape formations printed using SLA technology	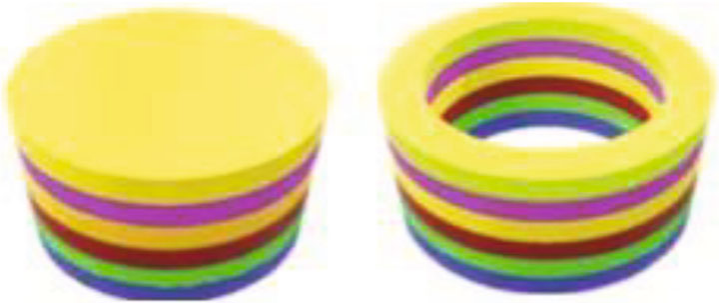	[39]
3D-printed dosage forms in radiator-like configurations	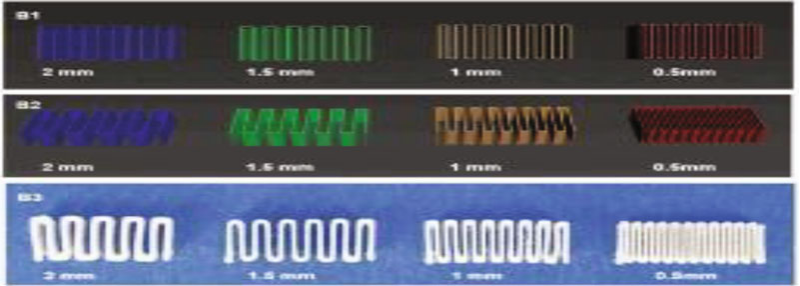	[15]
Scanning and computer-aided design (CAD) of wound area and printing of individualized wound dressings	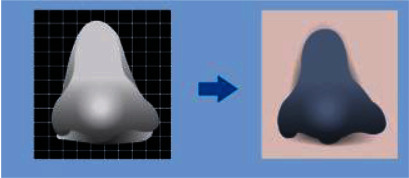	[40]
Printed intrauterine system (IUS)	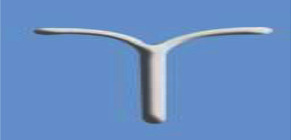	[41]

## Data Availability

The authors have nothing to report.
